# Cervical *Mycobacterium genavense* infection in a patient with lymphadenitis and previously unknown anti-IFN-γ IgG autoantibodies

**DOI:** 10.1007/s15010-025-02539-3

**Published:** 2025-05-16

**Authors:** Ioannis Michaelides, Stilla Bauernfeind, Uwe Kölsch, Florian Hitzenbichler, Christopher Bohr, Constantin A. Hintschich

**Affiliations:** 1 Department of Otorhinolaryngology, Regensburg University Hospital, Regensburg, Germany; 2 Department of Infection Prevention and Infectious Diseases, Regensburg University Hospital, Regensburg, Germany; 3 Department of Immunology, Labor Berlin – Charité Vivantes GmbH, Berlin, Germany; 4 Department of Immunology, Institute of Medical Diagnostic – Berlin Potsdam GbR, Berlin, Germany; 5 Department of Ophthalmology, University Hospital of Munich (LMU), Munich, Germany

**Keywords:** *Mycobacterium genavense*, nAIGA, IL-2, IFN-gamma, Lymphadenitis

## Abstract

**Background:**

Infections with atypical mycobacteria are rare and sometimes difficult to correctly diagnose. In many cases underlying diseases such immune deficiency can promote these infections.

**Case presentation:**

A 43-year-old male of Southeast Asian origin presented to our tertiary care hospital with persistent cervical lymphadenopathy non-responsive to antibiotics. Imaging suggested malignancy, but a biopsy did not confirm this suspicion. PCR diagnostics identified Mycobacterium genavense and further immunological testing revealed an acquired adult-onset immunodeficiency due to neutralizing anti-IFN-γ autoantibodies (nAIGA), explaining both the current infection and previous pleural empyema. The patient responded well to triple antimycobacterial therapy, with no recurrence or novel infection after almost two years.

**Conclusions:**

Our case highlights the importance of considering underlying immunodeficiencies and the patient’s geographic origin in the diagnosis of rare infections.

A 43-years-old male patient presented to our tertiary care hospital with a three-week history of left sided cervical swelling. The swelling was progressive despite a previous empirical antibiotic treatment with amoxicillin/clavulanic acid. Preexisting conditions were arterial hypertension, obstructive sleeping apnea and psoriasis, and a recent diagnosis of Graves’ disease. Five months earlier, a pleural empyema was drained and lung segments 9/10 were resected and the patient was treated with amoxicillin/clavulanic acid. The histopathological report showed chronic inflammation and fibrosis. The patient was born and raised in the Philippines and was adopted by a German family at the age of 9 years.

Besides an indurated swelling of the left neck, the clinical examination of ear, nose, and throat was normal. Ultrasound and CT-scan showed a suspicious mass with a diameter of 4 cm and extensive bilateral cervical and thoracal lymphadenopathy (Fig. 1a, b). Standard laboratory testing revealed an elevated white cell count (14.8/nl [normal range: 3,98 − 10/nl]) and C-reactive protein (30 mg/l [ normal range < 5 mg/l]).

**Fig. 1:**
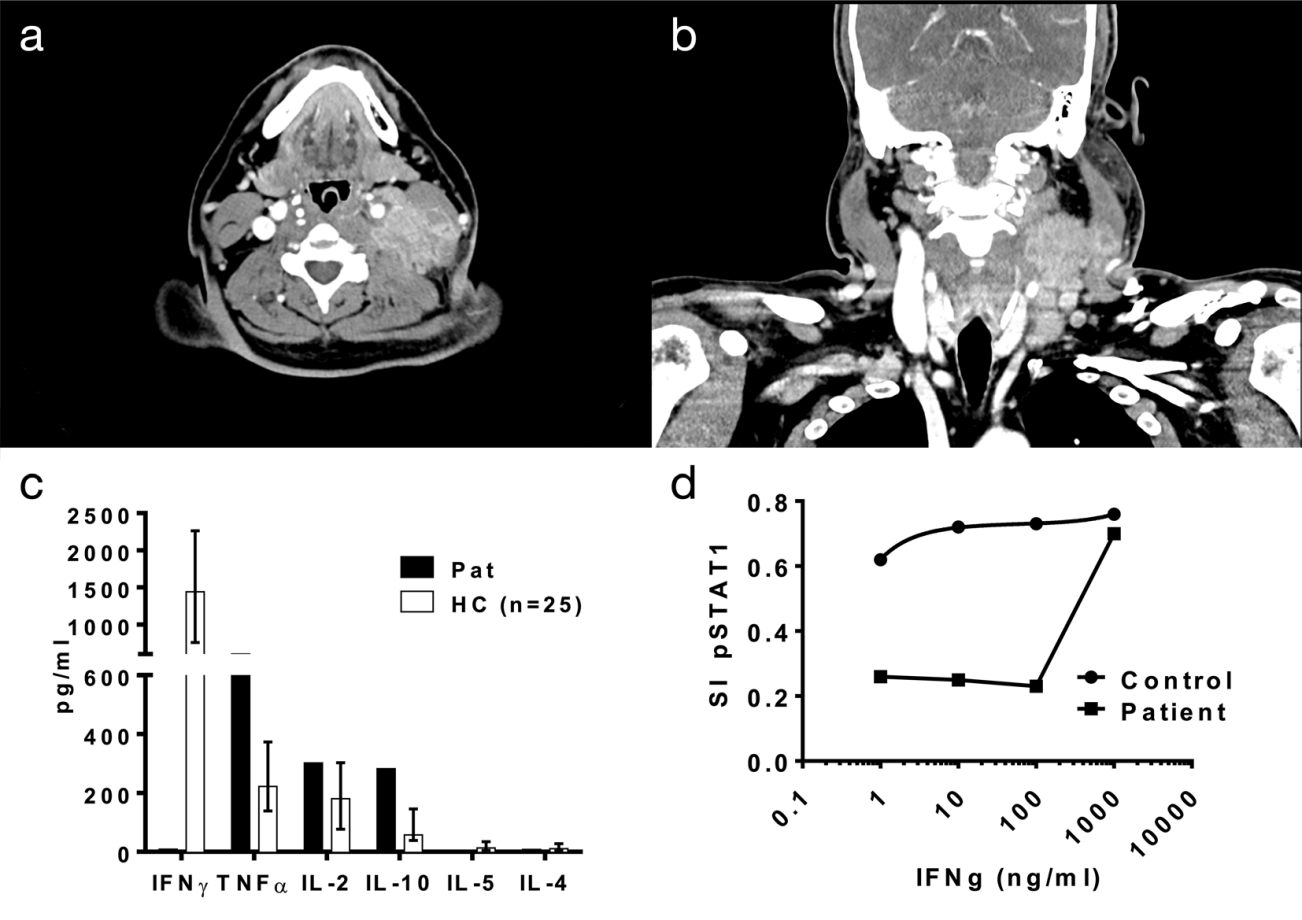
(**a**, **b**) CT scan showing cervical lesion at the patient’s admission. (**c**) IFN-γ production was not detectable in patient’s monocytes upon polyclonal stimulation with ConA (2.5 µg/ml for 24 h), whereas other cytokines were detectable as in controls with CBA-bead assay (BD, New York/USA). (**d**) pSTAT1 assay demonstrating complete inhibition of IFN-γ in the physiological range by anti-IFN-γ IgG autoantibodies; *p < 0.05. Methods in [11]

As our findings suggested a high probability for malignancy, a core needle biopsy of the cervical lesion was performed. The histopathological examination revealed an ulceration and necrosis zone infiltrated through granulocytes. Additionally, lymphoplasmacytic infiltration and chronic granulomatous fibrous lymphadenitis were observed, with no signs of malignancy or a specific infection. A Tuberculosis T-SPOT.TB^®^ ELISpot was negative. An open biopsy brought no additional histopathological information. In the microbiological workup, 16 S rDNA-PCR was positive for Mycobacteria spp. and sequencing analysis using SmartGene 2001–2020 to reported strains of *M. genavense* (LC533965 and AB973496). Cultures for mycobacteria for six weeks in fluid and solid media remained negative.

Due to the common association between *Mycobacterium genavense* infection and immune deficiency conditions, tests for lymphocyte subsets, immunoglobulins, and human immunodeficiency virus (HIV) were added but revealed no pathological findings. Given the association of atypical mycobacterial infections with interleukin 12 (IL-12)/interferon γ (IFN-γ) pathway disorders described predominantly in Asian people [1, 2], the Philippine origin of our patient induced screening for those defects. Here, in vitro IFN-γ production could not be measured upon stimulation of patient-derived monocytes (Fig. 1c). Further testing revealed high titer neutralizing anti-IFN-γ IgG autoantibodies (nAIGA) completely blocking IFN-γ within the physiologic range (Fig. 1d).

We initiated an empirical triple therapy with clarithromycin (500 mg 1-0-1), ethambutol (1500 mg 1-0-0), and rifabutin (300 mg 1-0-0), adjusted to the patient’s body weight (98 kg). Follow-ups by MRI and neck ultrasound showed a slow but continuous remission, and 20 months after diagnosis the antimycobacterial therapy was terminated. Up to now, a 23-month follow-up has shown no recurrence of mycobacterial infection or new infections caused by other facultative or obligate intracellular pathogens since the initial anti-IFN-γ IgG autoantibody titer was not very high and could inhibit IFN-γ response in some assays only partially.

## Discussion

*Mycobacterium genavense* is an opportunistic nontuberculous mycobacterium with a tropism for the intestinal tract first described in 1992 [3]. Initially known for life-threatening infections in people living with HIV, associations with other immune deficiencies have been described in recent years [4]. Cases in immunocompetent hosts are extremely rare. A recent meta-analysis has included only two cases of immunocompetent adults which may be attributed to mislabeling resulting from a failure to diagnose rare underlying immunodeficient conditions [4].

One important immune defense mechanism for the elimination of intracellular pathogens is signal amplification by the IL-12/IFN-γ circuit. Therefore, deficits in this pathway, such as the acquired adult-onset immunodeficiency nAIGA, predispose individuals to mycobacterial infections, but also other intracellular pathogens such as Salmonella or Toxoplasma. Possibly, the previous pleural empyema may have already occurred within the context of nAIGA [5]. Additionally, such conditions can also contribute to the pathogenesis of autoimmune diseases and tumors [6], suggesting a potential link to the patient’s diagnosis of Graves’ disease. His Philippine origin led us on the right track, as nAIGA predominantly affects individuals of Southeast Asian descent and appears to be associated with HLA alleles DRB*16:02 and DRB*05:02 [7, 8], that are expressed to a higher extend in the Asian population. Since we only tested T-SPOT.TB^®^ ELISpot with an unsuspicious negative result we did not detect the hint to anti-IFN-γ autoantibodies earlier. A parallel use of QuantiFERON-TB Gold Plus^®^ assay with no measurable IFN-γ production even not in the PHA-stimulated positive control of this QuantiFERON-TB Gold Plus^®^ assay could have been suspicious for anti-IFN-γ autoantibodies before. Furthermore, it is worth mentioning that using a Quantiferon^®^ assay could lead to an earlier discovery of the autoantibodies as through their presence no signal would be registered even in the positive control.

The treatment of infections with *M. genavense* generally consists of a combined antimycobacterial therapy for several months. The most common regimen includes a macrolide (e.g. clarithromycin, azithromycin), a rifamycin (e.g. rifabutin), and ethambutol [4]. In cases of an underlying IFN-γ deficiency and an inadequate response to antimycobacterial therapy, an additional immunostimulatory treatment with granulocyte-macrophage colony-stimulating factor (GM-CSF), plasmapheresis, cyclophosphamide, or Rituximab has to be considered. When IFN-γ antibodies are absent at all a treatment with IFN-γ should be considered as well [9, 10]. However, this was not necessary in our case.

This case illustrates how a rare mycobacterial infection can lead to the diagnosis of an acquired adult-onset immunodeficiency that can also provide an explanation for other health conditions. It further emphasizes the necessity of always considering patients’ origins, as this can provide crucial clues.

## Data Availability

No datasets were generated or analysed during the current study.
